# Elterliche Sexualaufklärung und sexuelles Risikoverhalten bei Töchtern und Söhnen: Befunde aus der Repräsentativbefragung „Jugendsexualität“

**DOI:** 10.1007/s00103-023-03783-4

**Published:** 2023-10-19

**Authors:** Nicola Döring, Roberto Walter, Sara Scharmanski

**Affiliations:** 1 Technische Universität Ilmenau, Institut für Medien und Kommunikationswissenschaft (IfMK), Ilmenau, Deutschland; 2 Bundeszentrale für gesundheitliche Aufklärung (BZgA), Köln, Deutschland; 3 Institut für Medien und Kommunikationswissenschaft (IfMK), TU Ilmenau, Ehrenbergstraße 29, 98693 Ilmenau, Deutschland

**Keywords:** Eltern, Sexuelle Bildung, Sexuelle Kommunikation, Sexuelle Sozialisation, Verhütungsaufklärung, Contraception education, Parents, Sex education, Sexual communication, Sexual socialization

## Abstract

**Hintergrund:**

Sexualaufklärung im Elternhaus soll laut Sozialisationstheorie zu mehr sexueller Handlungskompetenz bei Jugendlichen führen. Aktuelle Daten für Deutschland fehlen jedoch.

**Ziel der Arbeit:**

Vor diesem Hintergrund war es Ziel der vorliegenden Studie, erstmals das allgemeine Sprechen über Sexualität im Elternhaus (Forschungsfrage 1, F1) sowie speziell die Verhütungsberatung durch die Eltern (F2) mit dem sexuellen Risikoverhalten der Jugendlichen in Verbindung zu setzen.

**Material und Methoden:**

Datengrundlage ist die 9. Welle der Repräsentativbefragung „Jugendsexualität“ der Bundeszentrale für gesundheitliche Aufklärung (BZgA). Analysiert wurden Daten aller sexuell aktiven 14- bis 17-jährigen Jugendlichen im Sample, von denen eigene Angaben zum Sexualverhalten sowie Angaben ihrer Eltern zum Aufklärungsverhalten vorliegen (*N* = 357). Zur Beantwortung der beiden Forschungsfragen wurden logistische Regressionsanalysen mit 4 zentralen Merkmalen des jugendlichen Sexualverhaltens gerechnet.

**Ergebnisse:**

Es zeigte sich, dass das Sprechen über Sexualität im Elternhaus bei Mädchen und Jungen positiv korreliert mit 1. dem erreichten Konsensalter beim ersten Geschlechtsverkehr, 2. einem positiven Erleben des ersten Geschlechtsverkehrs, 3. einem zuverlässigen Verhütungsverhalten und 4. einer geringen Anzahl an Sexualpartner*innen (F1). Das gleiche Ergebnismuster ergab sich für die Verhütungsberatung durch die Eltern (F2).

**Diskussion:**

Die positiven Zusammenhänge zwischen elterlicher Sexualaufklärung und risikoärmerem jugendlichen Sexualverhalten gilt es hinsichtlich der zugrunde liegenden Kausalmechanismen genauer zu untersuchen.

## Abstract (en)

**Background:**

According to socialization theory, parental sex education should lead to increased sexual literacy in adolescents. However, current data for Germany is lacking.

**Research aim:**

Against this backdrop, the aim of the present study was to link for the first time the general conversations about sexuality in the family (research question 1, RQ1), and specifically parental contraception education (RQ2), with the sexual risk behavior of adolescents.

**Materials and methods:**

The database is the ninth wave of the representative survey “Youth Sexuality” conducted by the Federal Centre for Health Education in Germany (BZgA). Data from all sexually active 14- to 17-year-old adolescents in the sample who provided information on their sexual behavior and whose parents reported on their sex education behavior were analyzed (*N* = 357). To answer the two research questions, logistic regression analyses were carried out with four central characteristics of adolescent sexual behavior.

**Results:**

The results showed that for both girls and boys conversations about sexuality in the family are positively correlated with (1) reached age of consent at first sexual intercourse, (2) a positive first sexual intercourse experience, (3) reliable contraception use, and (4) a low number of sexual partners (RQ1). The same pattern of results emerged for contraception education by parents (RQ2).

**Discussion:**

The positive correlations between parental sex education and less risky adolescent sexual behavior need to be further investigated with respect to the underlying causal mechanisms.

## Hintergrund

Unter *Sexualaufklärung* im Allgemeinen und *Verhütungsberatung* im Speziellen versteht man die informelle oder formale Bereitstellung von sexuellen Informationen, einschließlich Informationen zur Empfängnis- bzw. Zeugungsverhütung, für Jugendliche und Erwachsene [1–3]. Der Begriff der Sexualaufklärung (engl. „comprehensive sexuality education“) wird in Forschung und Praxis heute weitgehend synonym mit dem Begriff der *sexuellen Bildung* (engl. „sex education“) verwendet. Bevölkerungsrepräsentative Umfragen wie die Trendstudie „Jugendsexualität“^1^ zeigen, dass Jugendliche in Deutschland heutzutage Sexualaufklärung und Verhütungsberatung von unterschiedlichen Ansprechpersonen (z. B. Eltern, Peers, Lehrkräften, Ärzt*innen) sowie aus diversen Medien (z. B. Fernsehen, Presse, Websites, soziale Medien) beziehen [2, 4, 5]. In den letzten Jahrzehnten ist die Bedeutung digitaler Medien im Bereich der Sexualaufklärung stark gestiegen [6–10]. Inzwischen will die große Mehrheit der jugendlichen Mädchen und Jungen (69 %) nach eigenen Angaben sexuelle Wissenslücken am liebsten via Internet schließen [4]. Damit haben digitale Medien als bevorzugte Quellen für sexuelle Informationen alle anderen Medien und auch alle persönlichen Ansprechpersonen überholt.

Gleichzeitig belegen die aktuellen Daten aus der 9. Welle der Studie „Jugendsexualität“ die anhaltend große Relevanz der elterlichen Sexualaufklärung: Die rund 3500 befragten 14- bis 17-jährigen Jugendlichen teilten im Untersuchungsjahr 2019 mehrheitlich (56 %) mit, dass sie ihre eigenen Eltern^2^ zu den „wichtigsten Personen bei der Aufklärung über sexuelle Dinge“ zählen [2]. Für Mädchen ist dabei die Mutter die zentrale Ansprechperson (61 %), viel seltener der Vater (6 %), während Jungen den Vater (39 %), dicht gefolgt von der Mutter (30 %), als ihre wichtigste Person der Sexualaufklärung sehen [2].

Die Trendstudie „Jugendsexualität“ zeigt seit 1980 eine Zunahme der schulischen^3^ und elterlichen Sexualaufklärung und Verhütungsberatung im Jugendalter [11]. Sehr aufschlussreich zur Einschätzung der Bedeutung elterlicher Sexualaufklärung sind ergänzend die Daten aus der 1. Welle der Erwachsenenstudie „Gesundheit und Sexualität in Deutschland“^4^ (kurz: GeSiD). Hier gaben rund 5000 bevölkerungsrepräsentativ ausgewählte 18- bis 75-jährige Erwachsene im Untersuchungszeitraum 2018/2019 Auskunft unter anderem darüber, welche Sexualaufklärung im Jugendalter sie erhalten hatten und wie zufrieden sie aus heutiger Sicht mit dieser Sexualaufklärung sind [12]. Es zeigte sich im Generationenvergleich eine immer stärkere Verbreitung elterlicher und schulischer Sexualerziehung: So gaben 17 % der 66- bis 75-Jährigen an, im Elternhaus sexuell aufgeklärt worden zu sein, gegenüber 62 % der 18- bis 25-Jährigen, was einer Steigerung um 45 Prozentpunkte entspricht [12]. Die Differenz bei der schulischen Sexualerziehung beträgt sogar 68 Prozentpunkte: 24 % der 66- bis 75-Jährigen erhielten schulische Sexualerziehung gegenüber 92 % der 18- bis 25-Jährigen [12]. Die Zufriedenheit mit der Sexualaufklärung im Jugendalter ist bei Erwachsenen in der Rückschau um mehr als ein 5-Faches größer, wenn Sexualaufklärung im eigenen Elternhaus stattgefunden hat [12].

Infolge elterlicher Sexualaufklärung verspricht man sich bei Jugendlichen mehr als die oben berichtete größere subjektive Zufriedenheit mit dem sexualbezogenen Informationsstand. Erwartet werden auch eine größere Handlungskompetenz, eine verbesserte sexuelle und reproduktive Gesundheit und damit einhergehend ein risikoärmeres Sexualverhalten. Bislang liegen jedoch für Deutschland keine aktuellen empirischen Daten vor, die das Aufklärungsverhalten der Eltern mit dem sexuellen Risikoverhalten ihrer jugendlichen Töchter und Söhne in Verbindung setzen. Die vorliegende Studie schließt diese Forschungslücke.

## Forschungsstand

Sozialisationstheoretisch wird davon ausgegangen, dass das Elternhaus eine wichtige Instanz ist, die Jugendlichen sexuelle Werte, Normen, Wissensbestände und Handlungskompetenzen vermittelt [13–17]. Diese Vermittlung erfolgt sowohl indirekt und nonverbal, etwa durch das Vorleben bestimmter Beziehungsmodelle und Geschlechterrollen, als auch direkt und verbal, etwa im persönlichen Gespräch und durch konkrete Handlungsempfehlungen, beispielsweise zur Nutzung bestimmter Methoden zur Verhütung ungewollter Schwangerschaften [2]. Dabei geben Eltern dann ihre eigenen sexualbezogenen Wissensbestände, Einstellungen und Moralvorstellungen an die Kinder weiter, meist mit der Intention, diese vor Gefahren zu schützen und eine positive sexuelle Entwicklung zu fördern. Studien weisen darauf hin, dass Eltern sich eine qualitätsvolle Sexualaufklärung für ihre Kinder wünschen und diese auch gern selbst leisten würden, sich aber oft schwertun, sexuelle Themen anzusprechen [18], sei es, weil sie eigene Wissenslücken bemerken oder ihnen die Kommunikationsfähigkeiten für das sensible und oft schambehaftete Thema fehlen [19].

Trotz der großen und möglicherweise wachsenden Bedeutung nonverbaler sexueller Sozialisation im Elternhaus [17] behandelt die bisherige Forschung vor allem die verbale Sexualaufklärung. Diese wird typischerweise durch qualitative Interviews oder quantitative Umfragen erfasst, selten auch durch Beobachtungen sexualbezogener Gespräche zwischen Eltern und ihren Kindern (z. B. [20]). Internationale Forschungsübersichten zeigen, dass die elterliche verbale Sexualaufklärung ein breites Spektrum von Themen aufgreift, sich vor allem aber auf Risikoprävention konzentriert [13, 14, 21, 22].

Neben der Beschreibung der elterlichen Sexualaufklärung interessiert sich die Forschung für deren Voraussetzungen, dazu gehören Beziehungsfaktoren (z. B. gutes Eltern-Kind-Verhältnis), aber auch Elternfaktoren wie Wissen, Einstellungen, Scham oder sexuelle Leidenschaft [23, 24].

Hinsichtlich der für die vorliegende Analyse relevanten Effekte elterlicher Sexualaufklärung auf das jugendliche Sexualverhalten haben frühere Studien (z. B. [25–27]) vor allem die folgenden 3 abhängigen Variablen im Sinne einer Risikoreduktion betrachtet:

1. höheres Alter bzw. erreichtes Konsensalter^5^ beim ersten Geschlechtsverkehr,
2. zuverlässigeres Verhütungsverhalten bezüglich Schwangerschaft und/oder sexuell übertragbarer Infektionen sowie
3. geringere Anzahl an Sexualpartner*innen.

Für alle 3 Outcome-Variablen konnten laut bisherigen Forschungsübersichten [13, 14, 26, 28, 29] positive Zusammenhänge mit dem Angebot und Umfang elterlicher Sexualaufklärung aufgezeigt werden. Gleichzeitig sind in diesen Übersichtsarbeiten aber auch Einzelstudien eingeschlossen, die keine oder gegenläufige Effekte zeigen. Dies wird in Verbindung gebracht mit Messproblemen (z. B. divergierende Angaben von Eltern und Jugendlichen zur familiären Sexualaufklärung), aber auch mit der Komplexität der sexuellen Kommunikation in Familien [30]. So scheint ein wichtiger Aspekt der elterlichen Sexualaufklärung und Verhütungsberatung darin zu bestehen, dass Eltern Wissen und Handlungsempfehlungen an ihre Kinder weitergeben. Hier kommt es dann aber stark darauf an, welche Botschaften das im Detail sind, wie sie vorgebracht werden und wie sie von den jugendlichen Kindern aufgenommen werden (z. B. als hilfreicher Rat oder als unerwünschte Einmischung und Kontrolle). Dementsprechend kann unpassende elterliche Sexualaufklärung auch negative Effekte haben (z. B. Reaktanz und Protestverhalten). Gleichzeitig darf sexuelle Kommunikation im Elternhaus nicht auf unidirektionale Informationsvermittlung von Eltern an ihre Kinder verkürzt werden. So scheint ein wichtiger Moderator familiärer Sexualaufklärungseffekte die Frage zu sein, inwiefern sie Jugendlichen den Raum gibt, ihre bisherigen sexuellen Erfahrungen vertrauensvoll und offen mit den Eltern zu teilen [31].

Die Dynamik sexueller Kommunikation in Familien wird durch weitere Aspekte, wie beispielsweise die Geschwister, mitbestimmt: Wer gleichgeschlechtliche ältere Geschwister hat, wendet sich in sexuellen Belangen eher an diese als an die Eltern [32, 33]. Nicht zuletzt spielen auch die geschlechtlichen und sexuellen Identitäten der Eltern wie der Kinder eine Rolle dabei, ob und wie offen in der Jugendphase der Kinder miteinander über Sexualität gesprochen wird [34].

Trotz der genannten methodischen Einschränkungen und theoretischen Komplexitäten [35] gibt es einen weitgehenden Konsens in der in diesem Abschnitt zitierten angloamerikanisch geprägten internationalen Forschung, dass elterliche Sexualaufklärung ein Schutzfaktor im jugendlichen Sexualverhalten darstellt [36].

## Forschungsziel

Vor dem Hintergrund des bisherigen Forschungsstandes ist es das Ziel der vorliegenden Studie, anhand aktueller und repräsentativer Daten aus der 9. Welle der Studie „Jugendsexualität“ Zusammenhänge zwischen dem Aufklärungsverhalten der Eltern (1. Sprechen über Sexualität im Elternhaus, 2. elterliche Verhütungsberatung) und einem risikoärmeren Sexualverhalten der Jugendlichen herzustellen. Dabei wird das „risikoärmere Sexualverhalten“ gemäß den in der oben beschriebenen Fachliteratur üblicherweise genutzten 3 zentralen Indikatoren erfasst, wobei zusätzlich auch das positive Erleben des sexuellen Debüts einbezogen wird. Damit ergeben sich 4 Indikatoren: 1. erreichtes Konsensalter beim ersten Geschlechtsverkehr, 2. positives Erleben des ersten Geschlechtsverkehrs, 3. zuverlässiges Verhütungsverhalten und 4. geringe Anzahl bisheriger Sexualpartner*innen. Demensprechend sind 2 Forschungsfragen zu beantworten:

### F1.

Gibt es positive Zusammenhänge zwischen dem allgemeinen Sprechen über Sexualität im Elternhaus und 4 zentralen Indikatoren eines risikoärmeren Sexualverhaltens bei Töchtern und Söhnen?

### F2.

Gibt es positive Zusammenhänge zwischen der Verhütungsberatung im Elternhaus und 4 zentralen Indikatoren eines risikoärmeren Sexualverhaltens bei Töchtern und Söhnen?

## Methode

### Untersuchungsdesign

Die vorliegende Arbeit ist eine Teilanalyse aus dem Datensatz der 9. Welle der Befragungsstudie „Jugendsexualität“, bei der es sich um eine bevölkerungsrepräsentative mündliche und schriftliche Wiederholungsbefragung bzw. Trendstudie [37] handelt, die durch das Forschungsinstitut Kantar/EMNID im Auftrag der Bundeszentrale für gesundheitliche Aufklärung (BZgA) umgesetzt wurde. Alle methodischen Details zum Untersuchungsdesign der 9. Welle sind an anderer Stelle veröffentlicht [38]. Das schließt auch die methodischen Details zur Sicherstellung hoher Repräsentativität durch bundesweite Auswahl der Zielpersonen gemäß zentralen Quotierungsvariablen samt einer statistischen Gewichtung des Samples angesichts disproportionaler Stichprobenanlage ein.

### Stichprobenbildung

Aus dem Gesamtsample der 2019 erhobenen 9. Welle der Studie „Jugendsexualität“ wurden alle Jugendlichen im Alter zwischen 14 und 17 Jahren in das Analysesample eingeschlossen (*N* = 357), die sexuell aktiv sind (d. h. schon mindestens einmal Geschlechtsverkehr hatten) und von denen Elternangaben zum Aufklärungsverhalten vorliegen (Tab. 1).

**Table 1:** 

Auswahlschritte	Fallzahl gesamt (gewichtet; ungewichtet)	Mädchen (gewichtet; ungewichtet)	Jungen (gewichtet; ungewichtet)
Unbereinigte Gesamtstichprobe^a^	6032; 6032	2873; 3604	3159; 2428
Eltern- und Jugenddaten paarig vorliegend^b^	1212; 2422	589; 1451	623; 971
Finale Stichprobe: Eltern- und Jugenddaten paarig vorliegend^b^, nur Jugendliche mit GV-Erfahrung	357; 707	178; 434	179; 273

*GV* Geschlechtsverkehr

^a^ Enthält Jugendliche und junge Erwachsene im Alter von 13–25 Jahren [38]

^b^ In die Auswahlschritte 2 und 3 wurden nur Jugendliche im Alter von 14 bis 17 Jahren einbezogen

### Instrument

Zur Beantwortung der beiden Forschungsfragen wurden als unabhängige Variablen 2 Aspekte des Aufklärungsverhaltens der Eltern erfasst, wobei die Angaben von den Eltern selbst stammen. Aufgrund der einleitend zitierten Geschlechtsspezifik familiärer Sexualaufklärung [2] wurde bei Töchtern jeweils die Mutter und bei Söhnen der Vater als wichtigste Person der Sexualaufklärung befragt:

1. *Thematisierung von Sexualität im Familienkreis*: Die Mütter (bei befragten Töchtern) und Väter (bei befragten Söhnen) bejahten oder verneinten, ob „im Familienkreis über Sexualität und Partnerschaft gesprochen“ wird (Item Q2_01 des Fragebogens für Eltern).
2. *Elterliche Verhütungsberatung*: Die Mütter (bei befragten Töchtern) und Väter (bei befragten Söhnen) bejahten oder verneinten, ob sie ihre Tochter oder ihren Sohn „schon einmal ausführlich über Möglichkeiten der Empfängnisverhütung beraten“ haben (Item Q3_01 des Fragebogens für Eltern).

Entsprechend dem bisherigen Forschungsstand wurden als abhängige Variablen 4 zentrale Indikatoren eines risikoärmeren Sexualverhaltens der Jugendlichen erfasst, wobei die Angaben von den sexuell aktiven Jugendlichen selbst stammen:

1. *Konsensalter beim ersten Geschlechtsverkehr*: Die Jugendlichen gaben in einem offenen Antwortfeld ihr Alter beim ersten Geschlechtsverkehr an (Item Q7_01 des Fragebogens für Jugendliche). Das Alter wurde dahingehend dichotomisiert, ob das gesetzliche sexuelle Konsensalter erreicht war (14–17 Jahre) oder nicht (11–13 Jahre).
2. *Positives Erleben des ersten Geschlechtsverkehrs*: Die Jugendlichen gaben an, ob sie „ihren ersten Geschlechtsverkehr“ als „etwas Schönes“ empfunden hatten oder nicht (Item Q7_06 des Fragebogens für Jugendliche).
3. *Zuverlässiges Verhütungsverhalten*: Die Jugendlichen gaben auf einer 5‑stufigen Ratingskala die Zuverlässigkeit ihres generellen Verhütungsverhaltens bezüglich einer Schwangerschaft an (Item Q8_12 des Fragebogens für Jugendliche). Die Ratingskala wurde dahingehend dichotomisiert, ob die Angaben auf ein zuverlässiges Verhütungsverhalten hinweisen („achte *immer *sehr genau darauf“, „achte *fast immer* darauf“) oder nicht („achte *in der Regel* darauf“, „achte *selten* darauf“, „achte *nie* darauf“).
4. Geringe Anzahl bisheriger Sexualpartner*innen: Die Jugendlichen gaben an, ob sie bisher in ihrem Leben mit 1, 2, 3 oder mehr als 3 Partner*innen „Geschlechtsverkehr/Sex hatten“ (Item Q6_16 des Fragebogens für Jugendliche). Die polytome Variable wurde dahingehend dichotomisiert, ob die Jugendlichen eine geringe Anzahl (1–2 Partner*innen) berichten oder nicht (3 und mehr Partner*innen).

Das Geschlecht der Eltern und der Jugendlichen wurde in der 9. Welle der Studie „Jugendsexualität“ jeweils binär erfasst.^6^ Auch alle anderen Variablen in der vorliegenden Analyse wurden als binäre Variablen erfasst bzw. wie oben beschrieben für die Auswertung dichotomisiert.

### Datenerhebung und Datenanalyse

Die Datenerhebung erfolgte auf der Basis informierter Einwilligung mittels mündlicher Befragung von Eltern und Jugendlichen, wobei die Jugendlichen intimere Fragen selbst am Computer ausfüllten (zu methodischen Details der Datenerhebung siehe [38]).

Die statistische Auswertung der erhobenen Daten erfolgte im Rahmen der vorliegenden Analyse mithilfe der Statistik-Software SPSS Version 28 (IBM Inc., Armonk, New York, USA). Im Zuge deskriptivstatistischer Auswertungen wurden absolute Häufigkeiten und Prozentwerte berechnet. Die inferenzstatistischen Analysen beinhalteten Konfidenzintervalle für Prozentwerte sowie univariate binäre logistische Regressionen. Das Signifikanzniveau wurde konventionell auf 5 % festgelegt.

## Ergebnisse

Der Ergebnisteil ist gemäß den beiden Forschungsfragen gegliedert, vorangestellt sind die Prävalenzangaben zu den betrachteten Variablen.

### Prävalenzen von elterlichem Aufklärungs- und jugendlichem Sexualverhalten

Die Prävalenzen für das elterliche Aufklärungsverhalten zeigen, dass laut Angaben der Eltern in der großen Mehrheit der Familien (rund 80 %) sowohl allgemein über Sexualität gesprochen wird als auch eine elterliche Verhütungsberatung erfolgt (Tab. 2). Gleichzeitig heißt das aber auch, dass jede*r 5. Jugendliche keine Sexualaufklärung im Elternhaus erhält.

**Table 2:** 

	Gesamtstichprobe *n *(gewichtet; ungewichtet)	Gesamtstichprobe % [95 % KI]	Mädchen % [95 % KI]	Jungen % [95 % KI]
**Aufklärungsverhalten der Eltern**				
*Thematisierung von Sexualität im Familienkreis*				
Nein	73; 139	20,4 [17,5–23,7]	17,0 [13,8–20,9]	23,8 [19,1–29,2]
Ja	284; 567	79,6 [76,3–82,5]	83,0 [79,1–86,2]	76,2 [70,8–80,9]
*Elterliche Verhütungsberatung*				
Nein	76; 142	21,2 [18,2–24,5]	16,3 [13,1–20,1]	26,1 [21,2–31,7]
Ja	281; 564	78,8 [75,5–81,8]	83,7 [79,9–86,9]	73,9 [68,3–78,8]
**Sexualverhalten der Jugendlichen**				
*Konsensalter beim erstem GV*				
Nein	19; 39	5,5 [4,0–7,5]	5,6 [3,7–8,2]	5,5 [3,3–8,9]
Ja (ab 14 Jahre)	332; 657	94,5 [92,5–96,0]	94,4 [91,8–96,3]	94,5 [91,1–96,7]
*Positives Erleben des ersten GV*				
Nein	88; 184	24,5 [21,5–27,9]	30,8 [26,6–35,3]	18,3 [14,1–23,3]
Ja („als etwas Schönes“)	270; 523	75,5 [72,1–78,5]	69,2 [64,7–73,4]	81,7 [76,7–85,9]
*Zuverlässiges Verhütungsverhalten*				
Nein	44; 82	13,3 [10,8–16,3]	9,0 [6,6–12,1]	17,7 [13,5–22,9]
Ja („immer“/„fast immer“)	290; 581	86,7 [83,7–89,2]	91,0 [87,9–93,4]	82,3 [77,1–86,5]
Geringe Anzahl bisheriger Sexualpartner*innen				
Nein	99; 191	27,7 [24,4–31,2]	25,4 [21,5–29,7]	29,9 [24,8–35,7]
Ja (1–2 Partner*innen)	258; 514	72,3 [68,8–75,6]	74,6 [70,3–78,5]	70,1 [64,3–75,2]

*GV* Geschlechtsverkehr, *KI* Konfidenzintervall

Die Prävalenzen für das jugendliche Sexualverhalten zeigen, dass laut eigenen Angaben der sexuell aktiven Jugendlichen überwiegend ein risikoarmes Sexualverhalten an den Tag gelegt wird. Die große Mehrheit hat das erste Mal Geschlechtsverkehr erlebt, nachdem das sexuelle Konsensalter von 14 Jahren erreicht wurde (95 %), und beschreibt es als schöne Erfahrung (75 %). Weiterhin verhütet die große Mehrheit zuverlässig (87 %) und hatte bisher lediglich 1–2 Sexualpartner*innen (72 %, Tab. 2). Es gibt dementsprechend aber auch eine nennenswerte Gruppe von Jugendlichen, deren Sexualverhalten durch größere Partner*innenzahl (28 %), unzuverlässiges Verhütungsverhalten (13 %) und sexuelle Aktivität vor dem Erreichen des Konsensalters (5 %) höhere Risiken birgt.

Weiterhin zeigen sich in den Prävalenzen Geschlechtsunterschiede: Anhand der nicht überlappenden 95 %-Konfidenzintervalle (KI) ist erkennbar, dass Mädchen (KI von 80–87 %) im Vergleich zu Jungen (KI von 68–79 %) statistisch signifikant häufiger Verhütungsberatung im Elternhaus erhalten. Gleichzeitig geben Mädchen auch statistisch signifikant häufiger als Jungen an, zuverlässig zu verhüten und das erste Mal Geschlechtsverkehr nicht als etwas Schönes erlebt zu haben (Tab. 2).

### Thematisierung von Sexualität im Familienkreis und jugendliches Sexualverhalten

Als Antwort auf Forschungsfrage 1 zeigte sich, dass das Sprechen über Sexualität im Elternhaus bei Jugendlichen jeweils – in absteigender Größe der Odds Ratios – statistisch signifikant positiv korreliert mit 1. dem erreichten Konsensalter beim ersten Geschlechtsverkehr, 2. einem positiven Erleben des ersten Geschlechtsverkehrs, 3. einem zuverlässigen Verhütungsverhalten und 4. einer geringen Anzahl bisheriger Sexualpartner*innen (Tab. 3).

**Table 3:** 

	Thematisierung von Sexualität im Familienkreis Ja % [95 % KI]	Univariate logistische Regression	
		OR [95 % KI]	*n* gewichtet
**Sexualverhalten der Jugendlichen**			
*Konsensalter beim ersten GV*			
Nein	45,4 [30,2–61,4]	1,00	19
Ja (ab 14 Jahre)	81,4 [78,1–84,4]	5,29 [2,67–10,47]*	332
*Positives Erleben des ersten GV*			
Nein	66,2 [58,7–72,9]	1,00	87
Ja („als etwas Schönes“)	83,9 [80,3–87,0]	2,67 [1,78–4,00]*	270
*Zuverlässiges Verhütungsverhalten*			
Nein	65,7 [54,4–75,5]	1,00	44
Ja („immer“/„fast immer“)	81,5 [77,9–84,5]	2,30 [1,36–3,87]*	290
Geringe Anzahl bisheriger Sexualpartner*innen			
Nein	73,9 [66,9–79,9]	1,00	99
Ja (1–2 Partner*innen)	82,1 [78,4–85,3]	1,62 [1,07–2,44]*	257

*GV* Geschlechtsverkehr, *KI* Konfidenzintervall, *OR* Odds Ratio

* Signifikante Odds Ratio *p* ≤ 0,05

Das gleiche Ergebnismuster wie für die Gesamtstichprobe zeigt sich deskriptiv- und inferenzstatistisch, wenn man die Daten für Mädchen und Jungen separat auswertet. Lediglich die Anzahl der bisherigen Sexualpartner*innen der Mädchen und der Jungen sowie das Verhütungsverhalten der Mädchen erreichten angesichts der kleineren Gruppengrößen nicht mehr die Signifikanzschwelle (Tab. 4).

**Table 4:** 

	Thematisierung von Sexualität im Familienkreis Ja % [95 % KI] – Mädchen	Univariate logistische Regression		Thematisierung von Sexualität im Familienkreis Ja % [95 % KI] – Jungen	Univariate logistische Regression	
		OR [95 % KI]	*n* gewichtet		OR [95 % KI]	*n* gewichtet
**Sexualverhalten der Jugendlichen**						
*Konsensalter beim ersten GV*						
Nein	37,8 [20,9–58,2]	1,00	10	53,0 [28,9–75,8]	1,00	10
Ja (ab 14 Jahre)	85,3 [81,5–88,5]	9,59 [4,00–22,99]*	165	77,6 [72,0–82,3]	3,07 [1,06–8,88]*	167
*Positives Erleben des ersten GV*						
Nein	72,3 [64,0–79,2]	1,00	55	56,1 [42,1–69,1]	1,00	33
Ja („als etwas Schönes“)	87,7 [83,5–91,0]	2,74 [1,64–4,58]*	123	80,8 [75,0–85,4]	3,29 [1,71–6,33]*	146
*Zuverlässiges Verhütungsverhalten*						
Nein	72,4 [55,7–84,5]	1,00	15	62,3 [47,4–75,2]	1,00	29
Ja („immer“/„fast immer“)	84,2 [80,1–87,6]	2,04 [0,93–4,46]	153	78,4 [72,2–83,5]	2,19 [1,10–4,38]*	137
Geringe Anzahl bisheriger Sexualpartner*innen						
Nein	79,3 [70,7–85,9]	1,00	45	69,3 [58,4–78,4]	1,00	53
Ja (1–2 Partner*innen)	84,5 [80,1–88,0]	1,42 [0,82–2,47]	132	79,6 [73,2–84,7]	1,73 [0,95–3,12]	125

*GV* Geschlechtsverkehr, *KI* Konfidenzintervall, *OR* Odds Ratio

* Signifikante Odds Ratio *p* ≤ 0,05

### Elterliche Verhütungsberatung und jugendliches Sexualverhalten

Als Antwort auf Forschungsfrage 2 zeigte sich, dass die Verhütungsberatung durch die Eltern ebenfalls statistisch signifikant positiv korreliert mit allen 4 ausgewählten Indikatorvariablen eines risikoärmeren Sexualverhaltens der Jugendlichen (Tab. 5). Dabei sind die Odds Ratios etwas geringer für die Verhütungsberatung als für das allgemeine Sprechen über Sexualität im Elternhaus.

**Table 5:** 

	Elterliche Verhütungsberatung Ja % [95 % KI]	Univariate logistische Regression	
		OR [95 % KI]	*n* gewichtet
**Sexualverhalten der Jugendlichen**			
*Konsensalter beim ersten GV*			
Nein	53,9 [38,0–69,1]	1,00	19
Ja (ab 14 Jahre)	80,1 [76,7–83,1]	3,44 [1,74–6,77]*	332
*Positives Erleben des ersten GV*			
Nein	70,2 [62,9–76,7]	1,00	88
Ja („als etwas Schönes“)	81,6 [77,8–84,8]	1,88 [1,25–2,81]*	269
*Zuverlässiges Verhütungsverhalten*			
Nein	65,5 [54,2–75,3]	1,00	44
Ja („immer“/„fast immer“)	81,6 [78,0–84,7]	2,34 [1,39–3,93]*	289
Geringe Anzahl bisheriger Sexualpartner*innen			
Nein	73,2 [66,1–79,2]	1,00	99
Ja (1–2 Partner*innen)	81,1 [77,3–84,4]	1,58 [1,05–2,37]*	257

*GV* Geschlechtsverkehr, *KI* Konfidenzintervall, *OR* Odds Ratio

* Signifikante Odds Ratio *p* ≤ 0,05

Das gleiche Ergebnismuster wie für die Gesamtstichprobe zeigt sich deskriptiv- und inferenzstatistisch, wenn man die Daten für Mädchen und Jungen separat auswertet. Lediglich die Anzahl der bisherigen Sexualpartner*innen der Mädchen und der Jungen, das Verhütungsverhalten der Mädchen und das Konsensalter der Jungen erreichten angesichts der kleineren Gruppengrößen nicht mehr die Signifikanzschwelle (Tab. 6).

**Table 6:** 

	Elterliche Verhütungsberatung Ja % [95 % KI] – Mädchen	Univariate logistische Regression		Elterliche Verhütungsberatung Ja % [95 % KI] – Jungen	Univariate logistische Regression	
		OR [95 % KI]	*n* gewichtet		OR [95 % KI]	*n* gewichtet
**Sexualverhalten der Jugendlichen**						
*Konsensalter beim ersten GV*						
Nein	54,6 [34,9–72,9]	1,00	10	53,3 [29,1–76,0]	1,00	10
Ja (ab 14 Jahre)	85,1 [81,3–88,3]	4,76 [2,03–11,17]*	166	75,1 [69,3–80,0]	2,64 [0,91–7,62]	166
*Positives Erleben des ersten GV*						
Nein	76,2 [68,3–82,7]	1,00	55	60,2 [46,1–72,7]	1,0	33
Ja („als etwas Schönes“)	87,0 [82,7–90,4]	2,09 [1,24–3,52]*	123	77,0 [70,9–82,1]	2,21 [1,16–4,24]*	146
*Zuverlässiges Verhütungsverhalten*						
Nein	75,9 [59,7–87,0]	1,00	15	60,1 [45,2–73,3]	1,0	29
Ja („immer“/„fast immer“)	85,0 [81,0–88,3]	1,80 [0,80–4,03]	153	77,7 [71,5–82,9]	2,31 [1,17–4,59]*	136
Geringe Anzahl bisheriger Sexualpartner*innen						
Nein	79,3 [70,7–85,9]	1,00	45	67,9 [57,0–77,2]	1,00	53
Ja (1–2 Partner*innen)	85,1 [80,8–88,6]	1,49 [0,86–2,60]	133	76,8 [70,2–82,3]	1,56 [0,88–2,79]	124

GV Geschlechtsverkehr, KI Konfidenzintervall, OR Odds Ratio

* Signifikante Odds Ratio *p* ≤ 0,05

## Diskussion

Die Diskussion gliedert sich in die Interpretation der Befunde, Limitationen und Fazit.

### Interpretation der Befunde

Es zeigte sich anhand der Daten der 9. Welle der bevölkerungsrepräsentativen Studie „Jugendsexualität“, dass Sexualaufklärung durch die Eltern bei sexuell aktiven Jugendlichen mit einem statistisch signifikant risikoärmeren Sexualverhalten einhergeht. Dies trifft auf alle 4 betrachteten zentralen Indikatoren eines risikoärmeren Sexualverhaltens zu – erreichtes Konsensalter beim ersten Geschlechtsverkehr, positives Erleben des ersten Geschlechtsverkehrs, zuverlässiges Verhütungsverhalten und geringe Anzahl der bisherigen Sexualpartner*innen. Dieses Ergebnismuster in der Gesamtstichprobe lässt sich replizieren für die 2 betrachteten Aspekte der elterlichen Sexualaufklärung (allgemeines Sprechen über Sexualität und gezielte Verhütungsberatung) sowie in den Teilgruppen der Töchter und Söhne. Damit stehen diese aktuellen Befunde aus Deutschland im Einklang mit sozialisationstheoretischen Erwartungen und früheren internationalen Studien, die elterliche Sexualaufklärung als Schutzfaktor hinsichtlich Risiken beim jugendlichen Sexualverhalten konzeptualisieren [13, 14, 26, 28, 29, 35].

Im Vergleich zu früheren Generationen in Deutschland [12] sowie zu Familien in den USA [25] ist elterliche Sexual- und Verhütungsaufklärung für deutsche Jugendliche mit einer Prävalenz von rund 80 % (Tab. 2) heute der Normalfall. Dabei hängen die von den Eltern berichtete allgemeine Sexualaufklärung (Tab. 3) sowie die Verhütungsberatung (Tab. 5) durchgängig positiv zusammen mit einem risikoärmeren Sexualverhalten ihrer Kinder im Alter von 14 bis 17 Jahren, wobei der engste Zusammenhang mit dem erreichten Konsensalter beim ersten Geschlechtsverkehr besteht. Vergleicht man die separaten Analysen für Töchter und Söhne (Tab. 4 und Tab. 6), so zeigt sich ein konsistentes Ergebnismuster im Sinne positiver Zusammenhänge für alle 4 betrachteten Variablen: erreichtes Konsensalter beim ersten Mal Geschlechtsverkehr, positives Erleben des ersten Geschlechtsverkehrs, zuverlässiges Verhütungsverhalten und geringe Anzahl an Sexualpartner*innen.

### Limitationen

Einschränkend ist jedoch zu betonen, dass es sich bei den vorliegenden Befunden um Zusammenhangsmaße handelt, die auf ganz unterschiedlichen Ursache-Wirkungs-Relationen basieren können. So könnte das risikoärmere Sexualverhalten der Jugendlichen tatsächlich kausal auf die elterliche Sexualaufklärung zurückgehen, die im besten Fall zu mehr Handlungskompetenz im Sinne von Risikovermeidung führt. Ebenso könnte es aber auch sein, dass Eltern, die mit ihren Kindern über Sexualität und Verhütung sprechen, sich in weiteren Dimensionen von Eltern unterscheiden, die keine Sexualaufklärung anbieten, und dass diese nicht untersuchten weiteren Dimensionen die eigentlichen Wirkfaktoren sind (z. B. allgemeines Vertrauensverhältnis zwischen Eltern und Kindern, Erziehungsstil der Eltern, soziales Milieu der Familien).

Neben dieser generellen Limitation, die alle korrelativen Outcome-Studien betrifft, sind einige weitere methodische Einschränkungen zu nennen. So basiert der Fragebogen der Studie „Jugendsexualität“ – wie es für sexualbezogene Mehrthemen-Umfragen (z. B. auch die eingangs erwähnte GeSiD-Studie) typisch ist – überwiegend auf Einzelitems, deren psychometrische Eigenschaften unbekannt sind. Verhütung wurde mit Bezug auf Schwangerschaften, nicht aber mit Bezug auf sexuell übertragbare Infektionen erfasst. Weiterhin beschränkte sich in der 9. Welle die Elternbefragung auf deutsche Eltern, sodass die Studie keine Aussagen über in Deutschland lebende Jugendliche mit Migrationsgeschichte zulässt. Ebenso sind durch die binäre Erfassung des Geschlechts keine Aussagen über Jugendliche möglich, die sich nicht als Mädchen oder Jungen identifizieren. Zudem war durch die zum Teil kleinen Subgruppen die statistische Teststärke bei den geschlechtsspezifischen Analysen eingeschränkt.

Letztlich ist noch auf konzeptueller Ebene anzumerken, dass die untersuchten Risikofaktoren jugendlichen Sexualverhaltens in Einklang mit dem Forschungsstand stehen und auch ihre fachliche Berechtigung haben. Dennoch ist nicht zu vernachlässigen, dass die Rede vom sexuellen „Risikoverhalten“ immer auch Gefahr läuft, normierend und stigmatisierend zu wirken. So kann beispielsweise eine höhere Anzahl an Sexualpartner*innen Ausdruck eines völlig normalen und gesunden jugendlichen Entwicklungsprozesses sein, wenn die Beteiligten ihre sexuelle Neugier oder Abenteuerlust einvernehmlich ausleben und dabei bewusst ungewollte Schwangerschaften und sexuell übertragbare Infektionen verhüten [39]. Gleichzeitig kann die Beschränkung auf nur eine*n Sexualpartner*in ein Risikofaktor sein, etwa wenn diese Fokussierung mit emotionaler oder sexueller Abhängigkeit einhergeht. Solche Nuancierungen sind mitzudenken, wenn in quantitativen Studien auf Aggregatebene vereinfachend mit ausgewählten Indikatoren für Risikoverhalten gearbeitet wird.

### Fazit

Der im vorliegenden Beitrag beschriebene bisherige Forschungsstand dokumentiert über die letzten Dekaden eine deutliche Zunahme verbaler Sexualaufklärung im Elternhaus. Dabei wird die elterliche Sexualaufklärung in der Fachliteratur als Schutzfaktor gegen Risiken jugendlichen Sexualverhaltens aufgefasst bzw. als Ressource für die sexuelle und reproduktive Gesundheit junger Menschen. Die in der vorliegenden Studie belegten statistisch signifikanten positiven Korrelationen zwischen elterlicher Sexualaufklärung und risikoärmerem Sexualverhalten der jugendlichen Töchter und Söhne stehen in Einklang mit diesen Annahmen, ohne jedoch kausale Wirkrichtungen erklären zu können. Experimentelle Felduntersuchungen und Interventionsstudien sind notwendig, um zu prüfen, ob eine gezielt umgesetzte elterliche Sexualaufklärung im Vergleich zu einer Kontrollgruppe wirklich ursächlich zu einer Risikominderung führt. Gleichzeitig sind weitere Beobachtungs- und Befragungsstudien sinnvoll, die Merkmale und Inhalte elterlicher Sexualaufklärung genauer rekonstruieren. Weiterhin sind evaluierte Praxismaßnahmen wünschenswert, die Eltern bei Bedarf noch besser befähigen, in unterstützender Weise mit ihren jugendlichen Kindern über deren sexuelle Erfahrungen, Fragen und Anliegen zu sprechen, am besten in einer Art und Weise, die von den Beteiligten nicht als belastend, peinlich oder unangenehm erlebt wird.

## Footnotes

1 Website der Studie „Jugendsexualität“: https://www.sexualaufklaerung.de/forschung/forschungsfelder/jugendsexualitaet/.

2 Mit „Eltern“ sind im vorliegenden Beitrag zusammenfassend sowohl die leiblichen Eltern als auch nicht-leibliche Eltern bzw. Erziehungsberechtigte gemeint.

3 Laut Urteil des Bundesverfassungsgerichts vom 21.12.1977 ist der deutsche Staat aufgrund seines Erziehungs- und Bildungsauftrags berechtigt, Sexualerziehung in der Schule durchzuführen. Die Details der schulischen Sexualerziehung sind in den Schulgesetzen und Lehrplänen der einzelnen Bundesländer geregelt. Der Sexualkundeunterricht an Schulen im Sinne des staatlichen Bildungs- und Erziehungsauftrags muss gleichzeitig das Erziehungsrecht der Eltern und das Persönlichkeitsrecht des Kindes beachten, also etwa eine Indoktrinierung vermeiden und Rücksicht nehmen auf verschiedene weltanschauliche und religiöse Überzeugungen. Die Teilnahme am Sexualkundeunterricht ist verpflichtend.

4 Website der GESID-Studie: https://gesid.eu/.

5 In Deutschland gelten Jugendliche ab 14 Jahre als sexuell mündig bzw. konsensfähig, bei Kindern bis 14 Jahren liegt dagegen ein Schutzalter vor, d. h., sexuelle Handlungen mit ihnen werden gemäß § 176 Strafgesetzbuch (StGB) als sexueller Missbrauch gewertet.

6 Für die 10. Welle der Studie „Jugendsexualität“ wird aktuell geprüft, ob und wie ein breiteres Spektrum an Geschlechtern in das Sample aufgenommen werden kann, und zwar mit ausreichenden Gruppengrößen für statistische Gruppenvergleiche.
